# Jonah and the Whale: A True Shark Story

**Published:** 1887-03-12

**Authors:** 


### JONAH AND THE WHALE: A TRUE SHARK STORY.

Mr. C. S. of South Kensington, who was for thirty years an Australian colonist, relates the following story, and vouches for its truth.

"I went out to Australia in 1840 in the ship *Ann Miln* of Dundee. When in the tropics we got dismasted, and lay tossing about for several days. Standing looking over the ship's side, I suddenly saw a large shark swimming about close to us, and immediately in its wake were several smaller fish, and a number of pilot fish. As soon as the sailors knew there was a shark near, they baited a hook with a piece of pork, and hung it out over the bows. The enemy swam cautiously up and smelt it in a very suspicious manner, as much as to say, 'You may be all right, but I don't like the look of you.' Just as we were expecting him to open his huge jaws and swallow it at a gulp, he turned away and left it without so much as a nibble.

"Then followed an operation which made me think I must be dreaming. Mrs. Shark (for it was a female) swam round under the stern, and every one of the smaller fish (excepting the pilot fish) suddenly disappeared whilst we were gazing at her. I rubbed my eyes to make sure that I was awake. No sooner was the operation complete, than the shark turned and swam back to the bows, where the pork was still suspended, and, without a moment's hesitation, gulped down the bait. She was hau'ed on deck and despa'ched with all convenient speed. We then set to work to make our *post mortem* examination without either coroner or jury. When the shark was opened the birds did not begin to sing, but a number of very lively-looking fish began to flounder about in what appeared to be otherwise a perfectly empty stomach. These were the smaller fish I had seen disappearing. They proved to be baby sharks. They averaged from eighteen inches to two feet in length, and about nine inches in circumference, and were no fewer than forty-two in number. They had the appearance of large and very energetic haddocks. I kept one of them alive in a tub of water for six weeks. A stomach or bag that could contain forty-two good-sized haddocks was of no mean capacity. When the young sharks were disposed of, it occurred to me that the prison which could contain them alive, might contain other and even larger creatures—such for example as a boy or man. To test the capacity of the mother shark's 'swallow,' I pulled its jaws asunder, and easily got my head and shoulders through the throat.

"This particular shark was about sixteen feet long. There are sharks of the white variety which reach the enormous length of thirty feet, and have therefore, a correspondingly wide throat. Such an animal could easily swallow and disgorge a man. People who have difficulties about the Bible-story of Jonah and the whale, should remember that the word rendered 'whale' by our translators really means 'large fish.' The passage would therefore read, 'The Lord had prepared a *large fish* to swallow up Jonah.' According to such a reading the man who, like me, has actually had his head and shoulders in the gullet of a shark, will see no difficulty in the much controverted story of Jonah. The white shark, which is the largest known, abounds off the Syrian coast."

